# Chromosomal analysis of non-small-cell lung cancer by multicolour fluorescent *in situ* hybridisation

**DOI:** 10.1038/sj.bjc.6601569

**Published:** 2004-02-17

**Authors:** H K Berrieman, J N E Ashman, M E Cowen, J Greenman, M J Lind, L Cawkwell

**Affiliations:** 1 Postgraduate Medical Institute, University of Hull, Cottingham Rd, Hull HU6 7RX, UK; 2 Hull York Medical School, University of Hull, Cottingham Rd, Hull HU6 7RX, UK; 3 Cardiothoracic Unit, Castle Hill Hospital, Hull HU16 5JQ, UK

**Keywords:** non-small-cell lung cancer, multicolour fluorescence *in situ* hybridisation, comparative genomic hybridisation, karyotype, structural genetic abnormalities, cytogenetic analysis

## Abstract

The cytogenetic abnormalities in non-small-cell lung cancer remain elusive due primarily to the difficulty in obtaining metaphase spreads from solid tumours. We have used the molecular cytogenetic techniques of multicolour fluorescent *in situ* hybridisation (M-FISH) and comparative genomic hybridisation (CGH) to analyse four primary non-small-cell lung cancer samples and two established cell lines (COR-L23 and COR-L105) in order to identify common chromosomal aberrations. CGH revealed regions on 5p, 3q, 8q, 11q, 2q, 12p and 12q to be commonly over-represented and regions on 9p, 3p, 6q, 17p, 22q, 8p, 10p, 10q and 19p to be commonly under-represented. M-FISH revealed numerous complex chromosomal rearrangements. Translocations between chromosomes 5 and 14, 5 and 11 and 1 and 6 were observed in three of the six samples, with a further 14 translocations being observed in two samples each. Loss of the Y chromosome and gains of chromosomes 20 and 5p were also frequent. Chromosomes 4, 5, 8, 11, 12 and 19 were most frequently involved in interchromosomal translocations. Further investigation of the recurrent aberrations will be necessary to identify the specific breakpoints involved and any role they may have in the aetiology, diagnosis and prognosis of non-small-cell lung cancer.

Lung cancer is the biggest cause of cancer mortality in the world and has one of the worst survival rates of any solid tumour. Around 75–80% of all cases of lung cancer diagnosed are non-small-cell lung cancer (NSCLC). Although our knowledge of the events involved in NSCLC at the gene level is increasing, little is known about the gross structural chromosomal changes involved in the disease, in common with the majority of solid tumours. This is due to the extreme difficulty in obtaining metaphase spreads of sufficient quality and quantity for cytogenetic analysis. Interpretation of the complex genomes characteristic of solid tumours is also technically difficult using conventional cytogenetic banding methods. Conversely, studies of haematological malignancies have identified specific chromosomal abnormalities that have proved to be of diagnostic and prognostic value (Mitelman, 2000).

Recently developed molecular cytogenetic techniques have increased the potential for karyotypic analysis of solid tumour samples. Comparative genomic hybridisation (CGH) allows screening of the entire genome in order to identify regions of gain or loss of DNA and map them according to chromosomal location (Kallioniemi *et al*, 1992). A major advantage of CGH is that tissue culture and preparation of metaphase spreads is not required as DNA extracted from a tumour specimen is used. Previous CGH studies of NSCLC cell lines and primary tumours have identified chromosomal regions that are commonly over- or under-represented, including gains of 1q, 3q, 5p, 7p and 8q and losses of 3p, 6q, 8p, 9p and 17p (Balsara *et al*, 1997; Petersen *et al*, 1997; Björkqvist *et al*, 1998; Speicher *et al*, 2000; Luk *et al*, 2001).

Multicolour fluorescent *in situ* hybridisation (M-FISH) is a karyotyping technique that uses whole chromosome paints (WCP) to label each chromosome a unique colour (Speicher *et al*, 1996). Use of this technique facilitates the karyotyping of tumour cells and allows the identification of marker chromosomes and complex chromosomal rearrangements that would previously have been uninterpretable. Additionally, the use of colour has opened up the field to noncytogeneticists. Despite these technological advances, acquisition of suitable metaphase spreads is still a problem and consequently much of the work on solid tumours with M-FISH (or the similar technique Spectral Karyotyping (SKY); Schröck *et al*, 1996) has been carried out using established cell lines. Although useful models for studying cancer, our previous analysis of independently cultured strains of a breast cancer cell line (Bahia *et al*, 2002) revealed unique abnormalities in each variant, suggesting that cell lines may not provide data as reliable and as clinically relevant as that obtained from primary tumour samples.

To date, 10 NSCLC cell lines (MGH7, A549, A427, BEN, Colo699, D51, D54, D97, D117 and DV-90) and just two primary tumour samples (both squamous cell carcinomas) have been analysed using M-FISH or SKY (Speicher *et al*, 2000; Gunawan *et al*, 2001; Luk *et al*, 2001). These studies, and previous studies using cytogenetic banding (Testa *et al*, 1994; Sundareshan and Augustus, 1996; Berker-Karaüzüm *et al*, 1998) have revealed highly complex cytogenetic changes in the majority of cases, with no common pattern of chromosome rearrangements. Despite this, some translocations have been reported by multiple authors: a t(3;19) was observed in four studies and t(1;19) and t(1;16) in three studies each (Sundareshan and Augustus, 1996; Speicher *et al*, 2000; Gunawan *et al*, 2001; Luk *et al*, 2001). Additionally, isochromosomes of 5p and 8q are frequently observed in NSCLC and are also seen in other solid tumours.

Here we present results from the analysis of four primary NSCLC tumour samples in short-term culture and two established NSCLC cell lines (COR-L23 and COR-L105) by M-FISH and CGH. To our knowledge, the karyotypes of these two cell lines have not previously been reported. The aim of our study was to examine the gross genomic changes present in order to identify structural and numerical alterations that may warrant further investigation using higher resolution molecular techniques. It is hoped that the identification of such changes may eventually lead to improvements in diagnosis and the identification of prognostic factors for NSCLC.

## MATERIALS AND METHODS

### Primary tumour samples

Samples of fresh lung tumour were obtained from patients undergoing surgery for NSCLC at Castle Hill Hospital. Informed consent was obtained from all patients and local ethical approval granted for the study. All tumour samples were processed on the day of surgery. Tissue samples were cut into pieces approximately 1 mm^3^ using sterile scalpels and a cell suspension obtained by digestion with 0.5 mg ml^−1^ type III collagenase (Sigma-Aldrich, Dorset, UK) at 37^°^C with 5% CO_2_ for 18–24 h. Cells were pelleted at 200 g for 4 min and resuspended in RPMI 1640 medium supplemented with 10% v v^−1^ foetal calf serum, 2 mM glutamine, 100 U ml^−1^ penicillin, 100 *μ*g ml^−1^ streptomycin and 2.5 *μ*g ml^−1^ fungizone (Life Technologies, Paisley, UK) and the suspension was transferred to 25 cm^2^ tissue culture flasks and incubated at 37°C with 5% CO_2_. Once cells began to grow in the flasks, the RPMI medium was changed for ACL-4 (Masuda *et al*, 1991). Flasks were monitored each day for cell growth and the medium changed as required (at least once a week). Characteristics of the primary tumour samples are given in Table 1

**Table 1:** Characteristics and colcemid treatment conditions of the four primary NSCLC samples

	**SCC-1**	**SCC-2**	**AC-1**	**AC-2**
Sex	Male	Male	Female	Male
Age (years)	71	76	53	69
Cancer site	LUL	RUL	RUL	LUL
Culture time	79 days	3 days	62 days	20 days
Colcemid	10 *μ*l, 10 *μ*g ml^−1^	100 *μ*l, 1 *μ*g ml^−1^	200 *μ*l, 10 *μ*g ml^−1^	150 *μ*l, 1 *μ*g ml^−1^
Treatment	16 h	16 h	3 h	16 h

SCC=squamous cell carcinoma; AC=adenocarcinoma; LUL=left upper lobe; RUL=right upper lobe.

.

### NSCLC cell lines

Two established NSCLC cell lines were acquired from the European Collection of Cell Cultures (ECACC, Wiltshire, UK) and were fully authenticated prior to distribution. The cell lines were COR-L23 (ECACC No. 92031919; Baillie-Johnson *et al*, 1985), a large cell carcinoma, and COR-L105 (ECACC No. 92031918), an adenocarcinoma, both established from pleural effusions from Caucasian males. Cell lines were cultured in RPMI 1640 medium supplemented with 10% v v^−1^ foetal calf serum, glutamine, penicillin, streptomycin and fungizone (as above).

### Preparation of metaphase spreads

Cell cultures were treated with colcemid (Life Technologies) prior to reaching confluence and incubated at 37°C. Cell lines received 200 *μ*l 10 *μ*g ml^−1^ colcemid for 1 h; treatment conditions for primary tumour samples are given in Table 1. Cells were harvested and treated with hypotonic solution (0.075 M KCl) for 20 min, fixed in three changes of fixative solution (3 : 1, methanol : acetic acid) and stored at −20°C overnight. Chromosome preparations were dropped onto cleaned, humidified standard microscope slides and stored overnight at room temperature. Slides were assessed using phase contrast microscopy to ensure the presence of metaphase spreads of suitable quality before performing M-FISH.

### M-FISH

M-FISH was performed as described previously (Ashman *et al*, 2002; Bahia *et al*, 2002) using the SpectraVysion™ Assay system (Abbott Laboratories, Maidenhead, UK). SpectraVysion consists of a 52-probe mix of WCPs labelled with different combinations of five fluorochromes (SpectrumGold, SpectrumRed, SpectrumGreen, SpectrumAqua and SpectrumFarRed). In total, 24 combinations of no more than three fluorochromes are used, resulting in a unique colour for each chromosome (22 autosomes and two sex chromosomes).

Before the probe was applied, metaphase preparations were enzymatically treated with RNase and pepsin to remove RNA molecules and cytoplasmic proteins. Exact conditions varied between preparations depending on the amount of residual cytoplasm. Slides were fixed in a 1% formaldehyde solution, dehydrated, denatured in 70% formamide/2 × saline sodium citrate (SSC) and dehydrated for a second time. A measure of 10 *μ*l of SpectraVysion probe was denatured, applied to the slides and incubated at 37°C for 72 h to allow the probe to hybridise to the chromosomes. Following hybridisation, slides were washed in 0.4 × SSC/0.3% NP-40 (Abbott) at 72°C for 2 min followed by 2 × SSC/0.1% NP-40 at room temperature for 30 s. Slides were air dried and counterstained with 10 *μ*l of 42 ng ml^−1^ 4,6-diamidino-2-phenylindole (DAPI) counterstain in antifade (Abbott).

**Figure 1:**
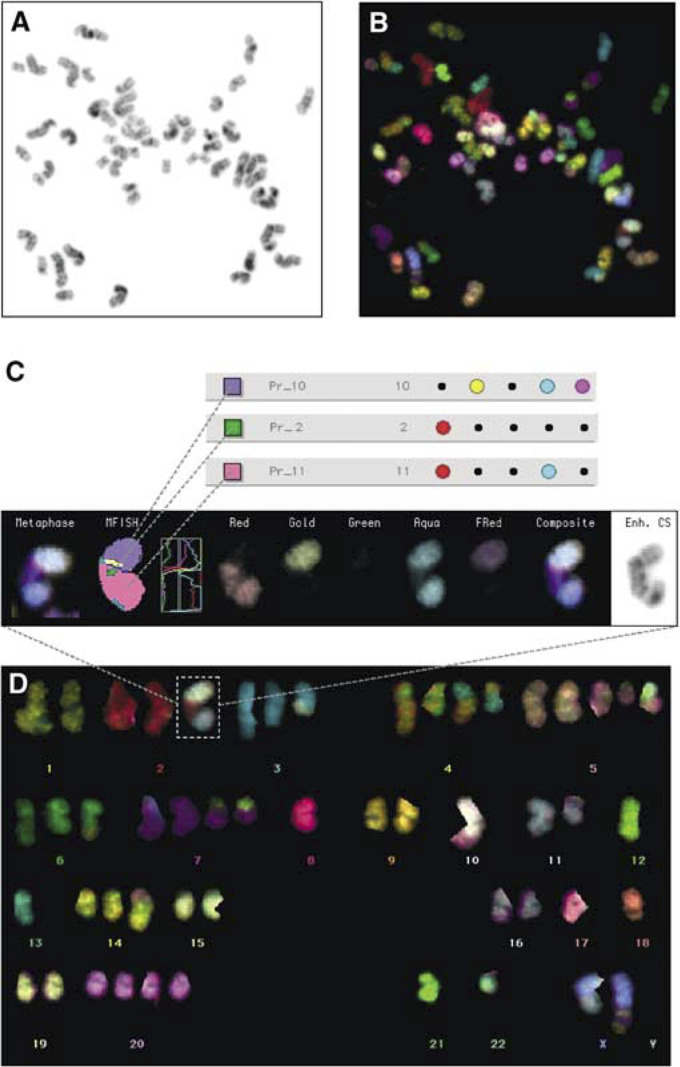
Analysis of a primary squamous cell carcinoma (SCC-1) by M-FISH: the metaphase spread stained with DAPI (**A**) and the M-FISH probe (**B**), an example of a translocation involving material originating from chromosomes 10, 2 and 11 (**C**) and the karyotype of the cell (**D**). The chromosome view shows (from left to right): the chromosome as it appears in the metaphase spread, the pseudocolours generated by the computer, a graph of the intensity of each fluorochrome along the chromosome axis, the individual colour planes (red, gold, green, aqua and far red), the composite image of all the colours and the chromosome stained with DAPI. This metaphase contains 51 chromosomes and is one of the seven spreads used in compiling the composite karyotype of the sample (see Table 2).

Images of metaphase spreads were captured using a Nikon® E800 epifluorescent microscope equipped with a Ludl 6 position filter wheel and a Photometrics Sensys™ cooled charge coupled device (CCD) camera. Six images were captured using filter combinations specific for each of the five fluorochromes and DAPI. All metaphases of suitable quality for analysis were captured. Spreads ideally contained long, well spread out chromosomes and did not contain large amounts of cell debris. Images were analysed using the Quips SpectraVysion software (Abbott) by examining each individual colour plane (Red, Gold, Green, Aqua and Far Red) and identifying the chromosomal material present by the combinations of fluorochromes observed. The analysis of a primary squamous cell carcinoma is shown in Figure 1

.

A composite karyotype was compiled for each sample using six to nine metaphase spreads. Following ISCN recommendations (ISCN 1995), rearranged chromosomes were included when two or more cells exhibited the same structural aberration. The modal number of each whole or rearranged chromosome was used in the composite karyotype. Translocations were reported in shortened ISCN format as derivative (der) chromosomes, but the break points could not be defined as identification using DAPI banding is difficult and requires cytogenetic expertise. Inversions and small deletions or amplifications are not identifiable using M-FISH.

### CGH

All CGH reagents were obtained from Abbott Laboratories and experiments were performed as described previously (Stafford *et al*, 1999). Briefly, 400 ng tumour DNA labelled with SpectrumGreen was combined with 200 ng reference DNA labelled with SpectrumRed and 20 *μ*g of Cot-1 blocking DNA. This probe mix was ethanol precipitated, resuspended in hybridisation buffer and hybridised to normal human metaphase chromosomes for 72 h at 37°C. Slides were washed in 0.4 × SSC/0.3% NP-40 followed by 2 × SSC/0.1% NP-40, air dried, and counterstained with 125 ng ml^−1^ DAPI in antifade. In total, 10 images of metaphase spreads were captured per sample using the same system as for M-FISH and analysed using the Quips CGH Analysis software (Abbott). Losses and gains of DNA sequences were scored whenever the ratio of red : green or green : red fluorescence exceeded thresholds established through hybridisations of differently labelled normal DNA samples (0.85 and 1.15, respectively).

## RESULTS

### M-FISH

The composite karyotype for each sample analysed by M-FISH (see Figure 1) is shown in Table 2

**Table 2:** Composite karyotype for each tumour sample and cell line

**Sample**	**Composite karyotype**
SCC-1 ♂	**48–57** 1 × 2, der(1)t(1;X;6), 2 × 2, der(2)t(10;2;11), 3 × 2, der(3)t(3;10), 4, der(?)t(4;13) × 2 (two different translocations), 5 × 2, del(5?q), der(?)t(5;22), ?i(5p), 6 × 2, der(6)t(1;6), 7 × 2, der(7)t(7;12), der(?)t(7;12), 8, der(8)t(Y;8), 9 × 2, der(10)t(5;10), 11, der(?11)t(11;16;11), 12 × 2, 13 × 2, 14 × 2, der(14)t(5;14), 15 × 2, 16 × 2, 17 × 2, 18 × 2, 19 × 2, 20 × 4, ?i(21q), 22, der(X)t(X;11), der(X)t(X;12;7;1) **[cp7]**
SCC-2 ♂	**37–48** 1 × 2, ?del(2p), der(2)t(2;9), ?del(3p) × 2, 4, der(4)t(4;5), 5, ?i(5p), 6, 7 × 2, 8, der(8)t(8;11), 9, der(9)t(5;9), 10, der(10)t(8;10;3), 11, der(?11)t(11;5;12;7), der(?11)t(11;5;14), der(11)t(11;18), 12, der(12), 13, i(13q), 14, der(14)t(14;18), 15 × 2, 16, der(16)t(16;19), ?del(17p) × 2, 18, der(?18)t(18;11;8;6), der(?18)t(6;18) × 2, 19, der(19)t(8;19), 20 × 2, 21 × 2, 22, der(22)t(5;22;1), X, Y **[cp8]**
AC-1 ♀	**70–90** 1 × 4, der(1)t(1;9), 2 × 2, der(2)t(20;6;2) × 2, der(2)t(2;5), der(2)t(2;3), der(2)t(2;4), 3 × 3, der(3)t(3;9), der(3)t(3;16), 4 × 2, der(4)t(4;12), der(4)t(4,13), 5 × 2, der(5)t(5;6), der(5)t(5;17), 6 × 3, der(6)t(6;11) × 2, 7 × 3, der(7)t(7;18) × 2, der(7)t(7;22) × 2, 8 × 2, der(8)t(13;8;13) × 2, 9, der(9)t(9;14;11) × 3, der(9)t(9;14) × 2, 11 × 4, 12 × 3, der(12)t(4;12), 14, der(15)t(5;15), der(15)t(15;17) × 2, der(16)t(16;21) × 2, 17, der(17)t(10;17), 18 × 2, 19 × 4, 20 × 4, der(21)t(5;21), 22 × 2, der(22)t(22;18;22;18), X, der(X)t(X;4) **[cp6]**
AC-2 ♂	**24–47** 1, der(?1)t(1;12;2), der(?1)t(6;1;14;5), der(2)t(7;2;21), der(2)t(2;15), 3, del(3p), der(3), der(4)t(4;10), der(4)t(5;19;5;19;4;5;11), der(4)t(7;19;5;19;4;6;5;11), der(5)t(5;17), der(6)t(1;6), der(6)t(6;15), i(6p), der(8)t(1;8), der(8)t(5;8), der(8)t(8;12), der(9)t(4;9), der(9)t(7;9), der(9)t(9;17), 10, der(10)t(10;15), der(10)t(10;19;17), der(11)t(5;11), der(12)t(12;8;12;5;19;11;19), der(12)t(3;12), der(15)t(8;15), 16, der(16)t(19;5;12;5;16;4;19;5;19;7), 17, 18, der(19)t(8;19), 20 × 3, der(?21)t(12;21), der(22)t(3;22), der(22)t(14;22), der(X)t(3;X), der(X)t(5;X) **[cp6]**
COR-L105 (AC) ♂	**18–39** der(1)t(1; 11), der(1)t(1;6), 2 × 2, der(3)t(3;21), der(3)t(3;X;4;X), 4, 5, 6, 7, **der(7)t(7;10)**, 8, 9, 10, **der(10)t(7;10)**, der(11)t(5;11), 12, 13, 14, der(14)t(8;14), der(14)t(12;14), 15, der(15)t(15;12;8), 16, der(16)t(3;16), 17, 18, 19, der(19)t(16;19), 20, 21, 22 **[cp9]**
COR-L23 (LCC) ♂	**97–105** l × 4, 2 × 4, 3 × 4, der(3), der(3)t(1;3), 4 × 4, 5 × 4, ?i(5p) × 2, 6 × 4, 7 × 4, 8 × 6, 9 × 4, 10 × 4, 11 × 6, 12 × 5, 13 × 4, 14 × 6, 15 × 4, 16 × 6, 17 × 4, 18 × 4, 19 × 4, 20 × 6, 21 × 4, 22 × 4, X × 2, der(X)t(X;15) × 2 **[cp6]**

Bold numbers at the beginning of each karyotype are the range of chromosome numbers for the spreads analysed, numbers in square brackets are the number of spreads used to compile the composite karyotype. The number of each chromosome or altered chromosome given is the modal number. Bold text represents translocations that may be reciprocal.

. In total, 93 different interchromosomal translocations were observed in the composite karyotypes from the six samples and 17 of these were observed in more than one sample: 14 in two samples and three in three samples (Table 3

**Table 3:** 17 translocations that were observed in more than one sample by M-FISH and the number of times they were observed primary tumours and cell lines

**Translocation**	**Primary tumour (*n*=4)**	**Cell line (*n*=2)**	**Total (*n*=6)**
t(5;14)	3^a^	0	3
t(1;6)	2	1	3
t(5;11)	2	1	3
			
t(3;10)	2^a^	0	2
t(5;22)	2^a^	0	2
t(7;12)	2^a^	0	2
t(5;6)	2^b^	0	2
t(5;17)	2^b^	0	2
t(4;5)	2	0	2
t(4;13)	2	0	2
t(5;12)	2	0	2
t(8;19)	2	0	2
t(X;3)	1	1	2
t(X;4)	1	1	2
t(3;16)	1	1	2
t(8;12)	1	1	2
t(16;19)	1	1	2

a Two squamous cell carcinoma samples.

b Two adenocarcinoma samples.

). Every chromosome was involved in at least one translocation, but six chromosomes (4, 5, 8, 11, 12 and 19) appeared to be involved particularly frequently (Figure 2

**Figure 2:**
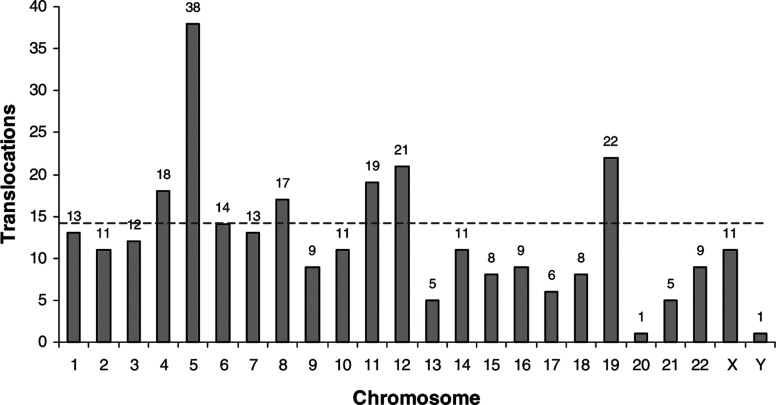
Graphical representation of the total number of interchromosomal translocations for each chromosome observed in the composite karyotypes for four primary NSCLC samples and two cell lines. The dashed line indicates an arbitary cutoff between the six chromosomes, which appear to be frequently involved in translocations and those which are involved less often.

). Chromosome 5 was the most frequently involved chromosome, with 38 translocations. Structures believed to be isochromosomes of 5p were observed in three samples and of 6p and 13q in one sample each.

Primary tumour samples had very complex karyotypes with many structural aberrations, whereas the two cell lines, especially COR-L23, were less complex and harboured only a few translocations (two in COR-L23 and 15 in COR-L105, compared with a mean of 32 in the primary tumour samples).

Numerical abnormalities were difficult to identify due to the complexity of the karyotypes and the involvement of the same chromosome in multiple rearrangements within a single karyotype. However, some recurrent numerical changes of whole chromosomes or chromosome arms were observed: a gain of chromosome 20 (above the ploidy level of the sample) in three samples, additional copies of 5p in two samples (some in the form of 5p isochromosomes), loss of the Y chromosome in three of the five male samples and deletions of 3p in two samples.

### CGH

CGH data for five of the six samples (AC-1, AC-2, SCC-2, COR-L23 and COR-L105) were available. The most common areas of loss and gain of DNA sequences and minimal regions of overlap are shown in Table 4

**Table 4:** Common regions of gain and loss of DNA sequences (including minimal regions of overlap) in primary tumour samples and cell lines observed by CGH

**Region**	**Primary tumour (*n*=3)**	**Cell line (*n*=2)**	**Total (*n*=5)**	**Minimal region of overlap**
*Gain*				
5p	3	2	5	p11–p13^a^
3q	2	2	4	q13.3–q25
8q	2	2	4	q23
11q	2	2	4	q13
2q	2	1	3	q22–q24
12p	1	2	3	p11.1–p11.2
12q	1	2	3	q21
				
*Loss*				
9p	3	2	5	p23
3p	2	2	4	p24–p25
6q	2	2	4	q25–qter
17p	2	2	4	p11.1–p12
22q	2	2	4	q13
8p	2	1	3	p21–p22
10p	2	1	3	p13
10q	2	1	3	q25–qter
19p	2	1	3	p13.2

a Four out of five samples (including two observed by M-FISH to have iso5p) showed gains of the entire 5p arm.

. Other regions of interest include 14q and 15q, where gains were seen in the two cell lines but not in any of the primary tumour samples (data not shown). The X and Y chromosomes were excluded from CGH analysis as samples and reference DNA were not sex-matched.

## DISCUSSION

There is little known about the gross chromosomal aberrations present in solid tumours, mainly due to the difficulty in obtaining metaphase spreads from such samples and the complex nature of the karyotypes, which makes analysis by banding patterns incredibly difficult. We have analysed four primary NSCLC tumours and two established NSCLC cell lines using modern molecular cytogenetic techniques that allow genetic aberrations to be elucidated, which would have been uninterpretable using conventional cytogenetic methods.

The karyotypes of the primary tumour samples were highly complex and contained many structural aberrations, including the involvement of some chromosomes in many different rearrangements within a single karyotype. The established cell lines showed generally less complex karyotypes, but structural alterations were still present. In total, 93 different interchromosomal translocations were observed in the six samples. Due to the genetic instability of cancer cells, many of these could merely be random rearrangements of unstable genetic material. However, 17 translocations were observed in either two or three samples, suggesting that combinations of specific chromosomes may occur nonrandomly. Of the translocations observed in two samples, only t(5;6), t(8;12) and t(8;19) have been reported by other authors (Gunawan *et al*, 2001; Speicher *et al*, 2000). Those translocations appearing in three samples are of particular interest, especially t(5;14), as this rearrangement was observed in three primary tumour samples, rather than in the established cell lines. In a recent study using M-FISH, Gunawan *et al* (2001) recorded the presence of two derivative chromosomes formed by a t(5;14) translocation in a primary squamous cell carcinoma. Two of the three samples in which we observed this translocation were also squamous cell carcinomas, although the samples size is far too small to be able to speculate as to whether this may be a histotype-related change. Gunawan *et al* (2001) also report a t(5;11) translocation, another of the rearrangements to be identified in three samples in this study. The remaining frequent translocation observed in our study, t(1;6), has also previously been reported by two authors (Luk *et al*, 2001; Speicher *et al*, 2000). Despite the small numbers of samples involved, the fact that these combinations of chromosomes have been reported in more than one study may imply that they could be important in the development of NSCLC. It is not possible to say whether the breakpoints involved in these common translocations are the same in each case. Further investigation of the chromosomes involved will be necessary to ascertain if this is so, and if specific genes located at the breakpoints are perturbed by the rearrangements.

Six chromosomes (4, 5, 8, 11, 12 and 19) were shown to be involved in rearrangements more often than the other chromosomes. Chromosome 5 was involved particularly frequently, with 38 interchromosomal translocations. In addition, the p-arm of this chromosome was the most frequently gained region as indicated by CGH. The possible formation of 5p isochromosomes observed by M-FISH is supported by the CGH data, as the two samples harbouring iso 5p for which CGH data are available show a gain of the entire p-arm. Formation of 5p isochromosomes would also require rearrangement of chromosome 5 material, thus further increasing the number of rearrangements involving this chromosome. Speicher *et al* (2000) reported frequent involvement of chromosomes 1, 2, 3, 11, 12 and 22 in intra- and interchromosomal changes. Chromosomes 5, 8 and 19 were observed by these authors to be involved less frequently, and chromosome 4 was involved infrequently in rearrangements. Our data therefore partially support those of Speicher *et al* (2000), and suggest that in addition to chromosome 5, chromosomes 11 and 12 may be of particular interest. The differences between the two studies may represent true differences between the tumours and cell lines analysed or may be a reflection of the small sample sizes used. This would only be resolved by a larger scale study. Although not all of the frequently rearranged chromosomes have been observed to participate in specific fusions with other chromosomes, the breakpoints involved warrant further investigation to discover if the same loci are involved each time.

The observation of 5p isochromosomes supports previous reports (Testa *et al*, 1994; Speicher *et al*, 2000; Luk *et al*, 2001). Polysomy of chromosome 20 has also been observed previously in NSCLC (Testa *et al*, 1994; Berker-Karaüzüm *et al*, 1998), and loss of the Y chromosome in males is known to be common in tumour cells.

The chromosome regions identified through CGH as being frequently gained are consistent with published results. Chromosome arms 3q, 5p and 8q, along with 1q and 7p, have been shown to be commonly over-represented in NSCLC. The minimal region of overlap on 8q in our study (q23) is the location of the *EBAG9/RCAS1* gene, which encodes a cancer cell-surface antigen implicated in immune escape and has recently been shown to be amplified in breast cancer (Tsuneizumi *et al*, 2001). A potentially important gene located within the minimal region of overlap on 5p (p11-p13) is *SKP2* at 5p13. This proto-oncogene encodes a protein which is associated with the cyclinA/cdk2 complex and is essential for transition into S-phase (Zhang *et al*, 1995). The minimal region of overlap observed on 11q (q13) is the site of the cyclin D1 gene which is an oncogene implicated in lung cancer. Gains of 11q have previously been reported in NSCLC by Petersen *et al*. (1997) and Luk *et al*. (2001). The final region gained in four samples was 3q (minimal region of overlap q13.3–q25). Gains of 3q have previously been shown to be important in lung cancer, especially 3q25–q27 in squamous cell carcinoma, suggesting that multiple genes within this region may be important for tumorigenesis (Kettunen *et al*, 2000).

The observation of losses on chromosomes 3p, 6q, 9p and 17p is also in agreement with the current body of literature relating to NSCLC (Balsara *et al*, 1997; Petersen *et al*, 1997; Björkqvist *et al*, 1998; Speicher *et al*, 2000; Luk *et al*, 2001). Losses of all these regions were observed in either four or five samples in this study. Chromosomes 9p and 17p are known to harbour the tumour suppressor genes *p16* and *p53*, respectively, but their locations do not correspond to the minimal regions of overlap identified in this study. Losses of 3p, including 3p25, are known to be frequent in lung cancer. The minimal region of loss observed here (3p24–p25) is in agreement with this. Potentially important genes within this region are retinoic acid receptor beta (*RARB*) at 3p24, which is a nuclear transcription factor thought to limit cell growth by regulating gene expression, and the von Hippel-Lindau (*VHL*) tumour suppressor gene at 3p25. Losses of 3p and 6q have previously been associated with the metastatic phenotype in both adenocarcinoma and squamous cell carcinoma of the lung (Petersen *et al*, 2000; Goeze *et al*, 2002). A further region found to be frequently lost in this study was 22q, with the minimal region of overlap at q13. The existence of putative tumour suppressor gene(s) in this region involved in breast and colorectal tumorigenesis has been proposed (Castells *et al*, 2000), but to our knowledge this has not previously been characterised in lung cancer.

In summary, we have demonstrated the power of M-FISH, in combination with CGH, in the study of primary NSCLC and established cell lines. The use of this colour-karyotyping technique has enabled the highly complex genomes of these samples to be analysed in far greater detail than would previously have been possible. Despite the small sample size, we have been able to identify certain chromosomes and chromosomal regions which are frequently involved in structural and numerical aberrations and which may therefore warrant further investigation. The use of higher resolution molecular genetic techniques will enable the specific breakpoints and genes involved in these aberrations to be identified. This in turn is likely to lead to an increased understanding of the aetiology and progression of NSCLC and yield information that can be used to improve patient diagnosis and prognosis.

## References

[bib1] Ashman JNE, Brigham J, Cowen ME, Bahia H, Greenman J, Lind M, Cawkwell L (2002) Chromosomal alterations in small cell lung cancer revealed by multicolour fluorescent *in situ* hybridization. Int J Cancer 102: 230–236 12397641 10.1002/ijc.10704

[bib2] Bahia H, Ashman JNE, Cawkwell L, Lind M, Monson JRT, Drew PJ, Greenman J (2002) Karyotypic variation between independently cultured strains of the cell line MCF-7 identified by multicolour fluorescence *in situ* hybridization. Int J Oncol 20: 489–494 11836559 10.3892/ijo.20.3.489

[bib3] Baillie-Johnson H, Twentyman PR, Fox NE, Walls GA, Workman P, Watson JV, Johnson N, Reeve JG, Bleehen NM (1985) Establishment and characterisation of cell lines from patients with lung cancer (predominantly small cell carcinoma). Br J Cancer 52: 495–504 2998422 10.1038/bjc.1985.220 PMC1977239

[bib4] Balsara BR, Sonoda G, du Manoir S, Siegfried JM, Gabrielson E, Testa JR (1997) Comparative genomic hybridization analysis detects frequent, often high-level, overrepresentation of DNA sequences at 3q, 5p, 7p and 8q in human non-small cell lung carcinomas. Cancer Res 57: 2116–2120 9187106

[bib5] Berker-Karaüzüm S, Lüleci G, Özbilim G, Erdoǧan A, Kuzucu A, Demircan A (1998) Cytogenetic findings in thirty lung carcinoma patients. Cancer Genet Cytogenet 100: 114–123 9428354 10.1016/s0165-4608(96)00422-0

[bib6] Björkqvist A-M, Husgafvel-Pursiainen K, Anttila S, Karjalainen A, Tammilehto L, Mattson K, Vainio H, Knuutila S (1998) DNA gains in 3q occur frequently in squamous cell carcinoma of the lung, but not in adenocarcinoma. Genes Chromosom Cancer 22: 79–82 9591638

[bib7] Castells A, Gusella JF, Ramesh V, Rustig AK (2000) A region of deletion on chromosome 22q13 is common to human breast and colorectal cancers. Cancer Res 60: 2836–2839 10850424

[bib8] Goeze A, Schluns K, Wolf G, Thäsler Z, Petersen S, Petersen I (2002) Chromosomal imbalances of primary and metastatic lung adenocarcinomas. J Pathol 196: 8–16 11748636 10.1002/path.1009

[bib9] Gunawan B, Mirzaie M, Schulten H-J, Heidrich B, Füzesi L (2001) Molecular cytogenetic analysis of two primary squamous cell carcinomas of the lung using multicolor fluorescence *in situ* hybridization. Virchows Arch 439: 85–89 11499845 10.1007/s004280100405

[bib10] ISCN (1995) An International System for Human Cytogenetic Nomenclature, Mitelman F (ed) Basel: S. Karger

[bib11] Kallioniemi A, Kallioniemi O-P, Sudar D, Rutovitz D, Gray JW, Waldman F, Pinkel D (1992) Comparative genomic hybridization for molecular cytogenetic analysis of solid tumours. Science 258: 818–821 1359641 10.1126/science.1359641

[bib12] Kettunen E, El-Rifai W, Björkqvist A-M, Wolff H, Karjalainen A, Anttila S, Mattson K, Husgafvel-Pursiainen K, Knuutila S (2000) A broad amplification pattern at 3q in squamous cell lung cancer – a fluorescence *in situ* hybridization study. Cancer Genet Cytogenet 117: 66–70 10700870 10.1016/s0165-4608(99)00146-6

[bib13] Luk C, Tsao M-S, Bayani J, Shepherd F, Squire JA (2001) Molecular cytogenetic analysis of non-small cell lung carcinoma by spectral karyotyping and comparative genomic hybridization. Cancer Genet Cytogenet 125: 87–99 11369051 10.1016/s0165-4608(00)00363-0

[bib14] Masuda N, Fukuoka M, Takada M, Kudoh S, Kusunoki Y (1991) Establishment and characterization of 20 human non-small cell lung cancer cell lines in a serum-free defined medium (ACL-4). Chest 100: 429–438 1650680 10.1378/chest.100.2.429

[bib15] Mitelman F (2000) Recurrent chromosome aberrations in cancer. Mutat Res 462: 247–253 10767636 10.1016/s1383-5742(00)00006-5

[bib16] Petersen I, Bujard M, Petersen S, Wolf G, Goeze A, Schwendel A, Langreck H, Gellert K, Reichel M, Just K, du Manoir S, Cremer T, Dietel M, Ried T (1997) Patterns of chromosomal imbalances in adenocarcinoma and squamous cell carcinoma of the lung. Cancer Res 57: 2331–2335 9192802

[bib17] Petersen S, Aninat-Meyer M, Schlüns K, Gellert K, Dietel M, Petersen I (2000) Chromosomal alterations in the clonal evolution to the metastatic stage of squamous cell carcinomas of the lung. Br J Cancer 82: 65–73 10638968 10.1054/bjoc.1999.0878 PMC2363206

[bib18] Schröck E, du Manoir S, Veldman T, Schoell B, Weinberg J, Ferguson-Smith MA, Ning Y, Ledbetter DH, Bar-Am I, Soenksen D, Garini Y, Ried T (1996) Multicolor spectral karyotyping of human chromosomes. Science 273: 494–497 8662537 10.1126/science.273.5274.494

[bib19] Speicher MR, Ballard SG, Ward DC (1996) Karyotyping human chromosomes by combinatorial multi-fluor FISH. Nat Genet 12: 368–375 8630489 10.1038/ng0496-368

[bib20] Speicher MR, Petersen S, Uhrig S, Jentsch I, Fauth C, Eils R, Petersen I (2000) Analysis of chromosomal alterations in non-small cell lung cancer by multiplex-FISH, comparative genomic hybridization, and multicolor bar coding. Lab Invest 80: 1031–1041 10908148 10.1038/labinvest.3780108

[bib21] Stafford ND, Ashman JNE, MacDonald AW, Ell SR, Monson JRT, Greenman J (1999) Genetic analysis of head and neck squamous cell carcinoma and surrounding mucosa. Arch Otolaryngol Head Neck Surg 125: 1341–1348 10604412 10.1001/archotol.125.12.1341

[bib22] Sundareshan TS, Augustus M (1996) Cytogenetics of non-small cell lung cancer: simple technique for obtaining high quality chromosomes by fine needle aspirate cultures. Cancer Genet Cytogenet 91: 53–60 8908167 10.1016/s0165-4608(96)00031-3

[bib23] Testa JR, Siegfried JM, Liu Z, Hunt JD, Feder MM, Litwin S, Zhou J-Y, Taguchi T, Keller SM (1994) Cytogenetic analysis of 63 non-small cell lung carcinomas: recurrent chromosome alterations amid frequent and widespread genomic upheaval. Gene, Chromosom Cancer 11: 178–194 10.1002/gcc.2870110307 7530487

[bib24] Tsuneizumi M, Emi M, Nagai H, Harada H, Sakamoto G, Kasumi F, Inoue S, Teruhisa K, Nakamura Y (2001) Overrepresentation of the *EBAG9* gene at 8q23 associated with early-stage breast cancers. Clin Cancer Res 7: 3526–3532 11705872

[bib25] Zhang H, Kobayashi R, Galaktionov K, Beach D (1995) p19Skp1 and p45Skp2 are essential elements of the cyclin A-CDK2 S-phase kinase. Cell 82: 915–925 7553852 10.1016/0092-8674(95)90271-6

